# Combined accelerated corneal collagen crosslinking and intrastromal Kerarings implantation for treatment of advanced superior keratoconus

**DOI:** 10.3205/oc000137

**Published:** 2020-02-27

**Authors:** Amr Mounir, Engy Mohamed Mostafa

**Affiliations:** 1 Sohag Faculty of Medicine, Ophthalmology Department, Sohag University, Sohag, Egypt

**Keywords:** superior keratoconus, Kerarings, corneal collagen crosslinking

## Abstract

**Purpose:** To report early results of combined accelerated corneal collagen crosslinking (CXL) and intrastromal Kerarings implantation by femtosecond laser in a case of advanced superior keratoconus.

**Methods:** The patient was a 27-year-old male with left eye grade 3 keratoconus with superior cone. He was diagnosed by Sirius Scheimpflug corneal topography (CSO, Florence, Italy). The right eye had previously been subjected to penetrating keratoplasty. The uncorrected visual acuity (UCVA) in the right eye (RE) was 1.2 LogMAR, best corrected visual acuity (BCVA) was 0.8 by a refraction of –7.50Ds –3.00Dc @12. The UCVA in the left eye (LE) was 1.2 LogMAR, BCVA was 0.9 by a refraction of –8.50 Ds –5.50Dc @169. Intrastromal Kerarings implantation by femtosecond laser was carried out by IntraLase (iFS, Abbott) along with accelerated transepithelial corneal collagen crosslinking by KXL system (Avedro, USA) in the same session. Follow-up was done for a period of 12 months after surgery.

**Results:** The patient was followed up for 12 months with improvement of visual acuity as regards UCVA and BCVA and improvement of corneal topographic parameters including keratometry and front and back elevations.

**Conclusion:** Combined accelerated corneal collagen crosslinking and intrastromal Kerarings implantation by femtosecond laser is an effective method in the treatment of this uncommon type of keratoconus.

## Introduction

Keratoconus is a noninflammatory disease in which the cornea thins and weakens. This biomechanical change leads to progressive deformation of the corneal optics with progressive visual impairment [1]. The keratoconic corneal apex is generally central or inferior in location [2]. Superior keratoconus is a rare entity of superior corneal ectasia. It can be detected either by clinical examination or corneal topographic examination [3]. There have been few reports of this uncommon form of keratoconus. Some of these cases have been subclinical types detected by corneal topography [4], [5], while others were with advanced stages of superior keratoconus with hydrops [6] or even induced corneal changes secondary to blepharoptosis [7]. The treatment strategy of keratoconus in general mainly includes strategies to improve visual acuity as rigid contact lens, corneal rings, penetrating keratoplasty and corneal collagen crosslinking, which is the only procedure that may slow down or stop keratoconus progression [8], [9]. In this case report, we are presenting the first reported results of combined accelerated corneal collagen crosslinking and intrastromal Kerarings implantation by femtosecond laser in a case of advanced superior keratoconus.

## Case description

A 27-year-old male was referred to our Center for Lasik and Corneal Surgeries with progressive loss of vision in the left eye. The uncorrected visual acuity in the right eye was 1.3 LogMAR corrected to 0.8 by a refraction of –7.50 Ds –3.00 Dc @12. The right eye had been previously subjected to corneal transplantation (penetrating keratoplasty). The indication of keratoplasty was not clearly known by the patient. The UCVA in the left eye was 1.2 LogMAR and best corrected visual acuity (BCVA) was 0.9 by a refraction of –8.50 Ds –5.50 Dc @169.

**Figure 1:**
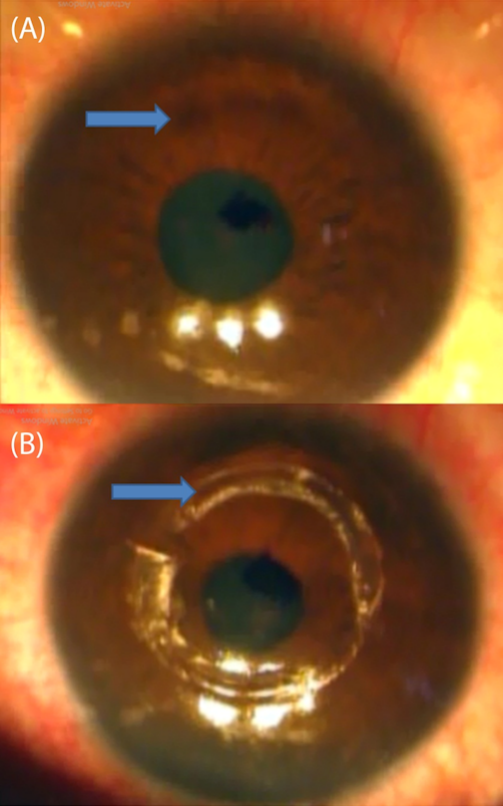
(A) Superior corneal protrusion without associated scarring or vascularization; (B) Two implanted Kerarings

Slit-lamp examination of the left eye revealed a superior corneal protrusion without associated scarring or vascularization (Figure 1A (Fig. 1)). There were no associated signs of endothelial dysfunction like increased stromal thickness or Descemet’s membrane folds or guttae. There were also no associated signs of corneal endotheliopathy like localized corneal edema or keratic precipitates or anterior segment reaction. The lens and the posterior segment did not show any remarkable findings.

Specular examination (Topcon SP-2000P, Topcon, Tokyo, Japan) of the left eye revealed normal corneal endothelium.

**Figure 2:**
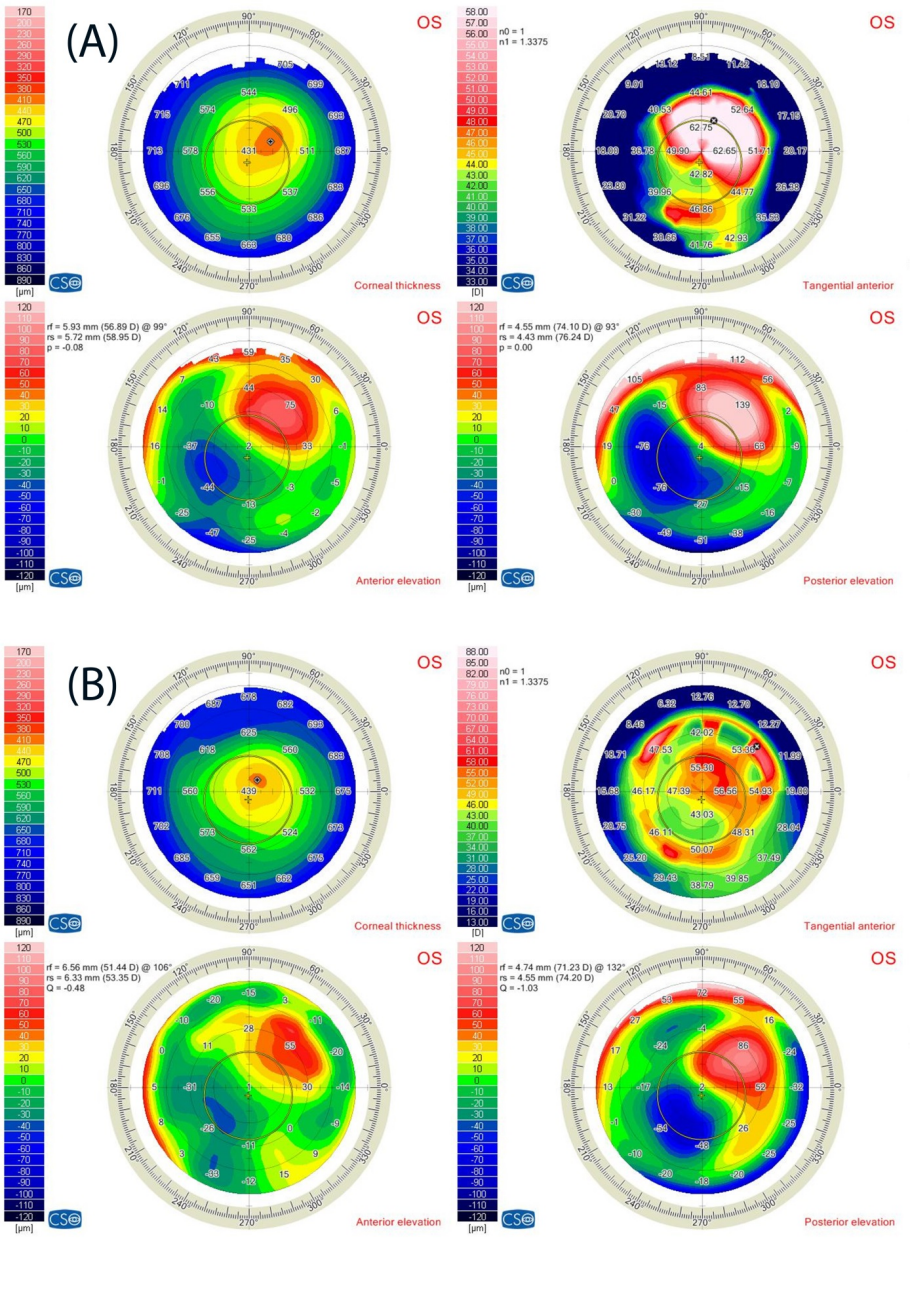
(A) Corneal tomography showed a cone in the superior part of the cornea. (B) Corneal tomography of the same eye after Kerarings implantation showed a marked decrease in the keratometry reading at the superior cone after 12 months.

Corneal tomography was done by Sirius Scheimpflug corneal tomography (CSO, Florence, Italy) for LE and revealed stage 3 keratoconus with superior cone. The corneal tomography showed a cone in the superior part of the cornea with Kmax: 62.7 D at the steepest point of the cone with corresponding high anterior and posterior elevation (75 μm and 139 μm) (Figure 2A (Fig. 2)). The apex of the cone was 3 mm superiotemporal from the corneal apex. Informed consent was obtained from the patient after having explained the treatment plan. This case report adhered to the tenants of the Helsinki declaration and was approved by the ethical committee of the center. The decision was to do left simultaneous intracorneal ring segments implantation with Kerarings (Mediphacos, Belo Horizonte, Brazil), followed by corneal collagen crosslinking in the same session. The tunnel for the rings was created with Advanced Femtosecond Laser (iFS, Abbott).

The procedure was started by marking a reference point for centration (Purkinje reflex). Femtosecond laser parameters for the corneal tunnel were:

- inner diameter: 5 mm,
- outer diameter: 5.9 mm,
- depth: 80% of thinnest central corneal thickness,
- incision site: at the axis of K2 (the steepest) corneal meridian, energy 2.00 mJ

The tunnel was opened using a blunt sinskey, then two Kerarings were implanted by the use of special ring forceps (Figure 1B (Fig. 1)). After Kerarings implantation, transepithelial accelerated corneal collagen crosslinking was done by the KXL^®^ System accelerated CXL (Avedro), the corneal surface was treated by the application of 0.25% riboflavin solution supplemented with BAC, EDTA, trometamol, hydroxypropyl-methylcellulose (ParaCell, Avedro) for 4.50 min, and 0.25% riboflavin solution (VibeX Extra, Avedro) for 6 min. Drops were applied every 90 s during the soak time; followed by 5.20 minutes accelerated CXL using the pulsed mode with 45 mW/CC power without corneal epithelial debridement. One drop of VibeX Extra was applied every 90 s during irradiation. The postoperative medication included topical antibiotics eye drops (e.g. Gatifloxacin 0.3% 5 times/day for one week), topical steroid eye drops (e.g. Prednisolone acetate 1% 5 times/day for one week, Lubricant eye drops and systemic non-steroidal anti-inflammatory drugs. The patient was followed up for 12 months. At the 12^th^ postoperative month, UCVA was 0.8 LogMAR and BCVA was 0.5 LogMAR by a refraction of –3.75 Ds –2.50 Dc @169.

Corneal tomography showed marked improvement in the form of decrease in the keratometry reading at the superior cone (Kmax: 56.65 D) and decrease in both anterior and posterior elevations (anterior elevation 55 μm, and posterior elevation 86 μm) (Figure 2B (Fig. 2)).

## Discussion

There are few reports presenting cases of superior keratoconus and most of them do not describe the visual rehabilitation of this uncommon type [10], [11], [12]. Weed et al. [4] reported the management of 2 cases of superior keratoconus with Tricare RGP with smaller diameter (8.8 mm) and an offset of 1 mm so that the distribution of bearing shifts superiorly with mild cornel apex touch. As regards our clinical decision, we excluded the liability of corneal endotheliopathy due to the absence of clinical signs of localized endothelial dysfunction. In addition, there was no abnormality detected in the corneal specular examination. 

In our case, keratoconus was of advanced stage (average K more than 52 Ds), the manifest refraction was of high spherical and cylindrical components. So we decided to do corneal collagen crosslinking as the mainstay of therapy, but the argument was whether to implant intracorneal rings to induce flattening or not, especially since all the available nomograms are designed for central and inferior cones, not the superior type.

The decision was to implant two Kerarings segments; 160/300 and 90/250. The larger one was implanted around the superior cone to produce the maximum flattening effect, while the smaller one was implanted at the opposite site with an offset of 1 mm from the pupil. Combined accelerated corneal collagen crosslinking and intrastromal Kerarings implantation has been found by many studies to be an effective procedure in treatment of keratoconus [13], [14], [15]. However, our main challenge in this case was that there is no consensus about the suitable treatment of this relatively rare type of keratoconus. The results of our combined procedure are promising for treatment of this uncommon type of keratoconus, but further studies are needed to achieve a suitable nomogram for intrastromal corneal rings implantation in keratoconus with superior cone location. 

## Conclusion

Combined accelerated corneal collagen crosslinking and intrastromal Kerarings implantation by femtosecond laser is an effective method in treatment of this uncommon type of keratoconus.

## Notes

### Competing interests

The authors declare that they have no competing interests.

### Ethical approval

All procedures performed involving a human participant were in accordance with the ethical standards of national research committee and with the 1964 Helsinki Declaration and its later amendments or comparable ethical standards.

### Informed consent

Informed consent was obtained from the patient after having explained the treatment plan.
